# Supplementary material for: Identification of photoperiod-induced specific miRNAs in the adrenal glands of Sunite sheep (Ovis aries)
Source: Front Vet Sci. 2022 Jul 22;9:888207. doi: 10.3389/fvets.2022.888207 (PMC9354845; doi:10.3389/fvets.2022.888207)
Supplement: Supplementary file 1 [file Data_Sheet_1.ZIP › Supplementary file/miRDeep2 file.zip!/miRDeep2 file/Know_Structure/SPLP42A2/oar-mir-10a.pdf]

miRBase precursor	: oar-mir-10a
Total read count	: 83034
oar-miR-10a read count	: 82504
remaining reads	: 530

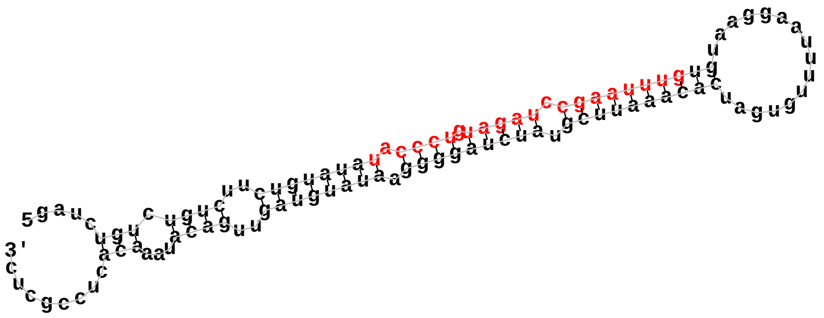

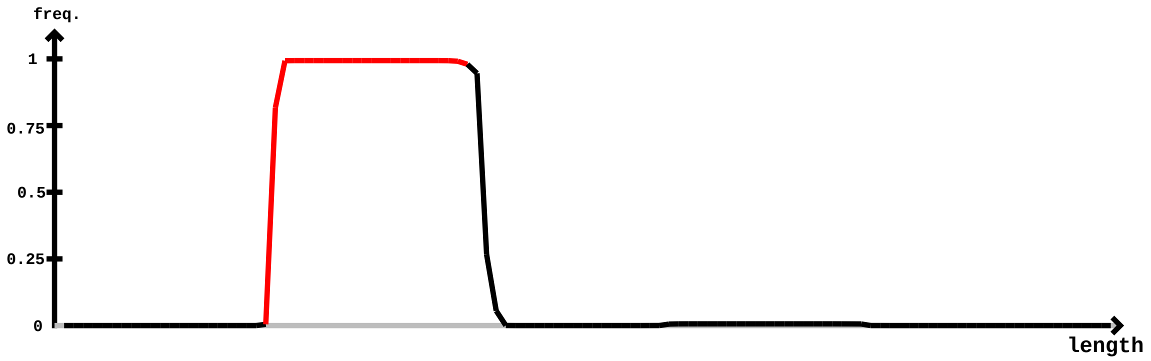


```
                                    oar-miR-10a
5'- gaucugucugucuucuguauauacccuguagauccgaauuuguguaaggaauuuugugaucacaaauucguaucuaggggaauauguaguugacauaaacacuccgcuc   -3'    exp
    ....(((.((((..((((((((.((((.(((((.((((((((((...............)))))))))).))))))))).))))))))..))))...)))........   reads      mm          sample
    ....................auacccuguagauccga........................................................................   1          0           seq
    ....................auacccuguagauccgaauu.....................................................................   3          0           seq
    ....................auacccuguagauccgaauuu....................................................................   26         0           seq
    ....................auacccuguagauccgaauuug...................................................................   77         0           seq
    ....................auacccuguagauccgaauuugu..................................................................   265        0           seq
    ....................auacccuguagauccgaauuugug.................................................................   22         0           seq
    .....................uacccuguagauccgaa.......................................................................   2          0           seq
    .....................uacccuguagauccgaau......................................................................   23         0           seq
    .....................uacccuguagauccgaauu.....................................................................   151        0           seq
    .....................uacccuguagauccgaauuu....................................................................   796        0           seq
    .....................uacccuguagauccgaauuug...................................................................   2363       0           seq
    .....................uacccuguagauccgaauuugu..................................................................   49484      0           seq
    .....................uacccuguagauccgaauuugug.................................................................   11989      0           seq
    .....................uacccuguagauccgaauuugugu................................................................   2618       0           seq
    .....................uacccuguagauccgaauuugugua...............................................................   8          0           seq
    .....................uacccuguagauccgaauuuguguaa..............................................................   1          0           seq
    .....................uacccuguagauccgaauuuguguaagg............................................................   1          0           seq
    ......................acccuguagauccgaau......................................................................   2          0           seq
    ......................acccuguagauccgaauu.....................................................................   32         0           seq
    ......................acccuguagauccgaauuu....................................................................   117        0           seq
    ......................acccuguagauccgaauuug...................................................................   370        0           seq
    ......................acccuguagauccgaauuugu..................................................................   6675       0           seq
    ......................acccuguagauccgaauuugug.................................................................   5479       0           seq
    ......................acccuguagauccgaauuugugu................................................................   1936       0           seq
    ......................acccuguagauccgaauuugugua...............................................................   16         0           seq
    ......................acccuguagauccgaauuuguguaa..............................................................   2          0           seq
    .......................cccuguagauccgaauuug...................................................................   1          0           seq
    .......................cccuguagauccgaauuugu..................................................................   16         0           seq
    .......................cccuguagauccgaauuugug.................................................................   17         0           seq
    .......................cccuguagauccgaauuugugu................................................................   9          0           seq
    .......................cccuguagauccgaauuugugua...............................................................   1          0           seq
    ........................ccuguagauccgaauuugu..................................................................   1          0           seq
    ........................ccuguagauccgaauuugugu................................................................   1          0           seq
    ...........................................................ucacaaauucguaucuagggg.............................   1          0           seq
    ...........................................................ucacaaauucguaucuaggga............................   2          0           seq
```

oar-miR-10a

```
gaucugucugucuucuguauauacccuguagauccgaauuuguguaaggaauuuugugaucacaaauucguaucuaggggaauauguaguugacauaaacacuccgcuc
............................................................cacaaauucguaucuaggggaau..........................   2   0   seq
.............................................................acaaauucguaucuagggga............................   1   0   seq
.............................................................acaaauucguaucuaggggaau..........................   12  0   seq
..............................................................caaauucguaucuagggg.............................   2   0   seq
..............................................................caaauucguaucuagggga............................   11  0   seq
..............................................................caaauucguaucuaggggaa...........................   14  0   seq
..............................................................caaauucguaucuaggggaau..........................   414 0   seq
..............................................................caaauucguaucuaggggaaua.........................   4   0   seq
..............................................................caaauucguaucuaggggaauau........................   1   0   seq
...............................................................aaauucguaucuagggga............................   1   0   seq
...............................................................aaauucguaucuaggggaa...........................   2   0   seq
...............................................................aaauucguaucuaggggaau..........................   54  0   seq
...............................................................aaauucguaucuaggggaaua.........................   1   0   seq
...............................................................aaauucguaucuaggggaauau........................   2   0   seq
................................................................aauucguaucuaggggaau..........................   1   0   seq
................................................................aauucguaucuaggggaauau........................   4   0   seq
```
